# Case Report: Endothelialized but unstable: late migration of a ventricular septal defect closure device

**DOI:** 10.3389/fcvm.2026.1807603

**Published:** 2026-04-20

**Authors:** Min-Jung Jan, Chung-Chi Wang, Sheng-Ling Jan, Wei-Li Liu

**Affiliations:** 1 Department of Medical Education, Tungs’ Taichung MetroHarbor Hospital, Taichung, Taiwan; 2 Cardiovascular Center, Taichung Veterans General Hospital, Taichung, Taiwan; 3 Children's Medical Center, Taichung Veterans General Hospital, Taichung, Taiwan; 4 Center for Quality Management, Taichung Veterans General Hospital, Taichung, Taiwan; 5 School of Medicine, Kaohsiung Medical University, Kaohsiung, and College of Medicine, National Chung Hsing University, Taichung, Taiwan; 6 Division of Pediatric Cardiology, Department of Pediatrics, Dalin Tzu Chi Hospital, Buddhist Tzu Chi Medical Foundation, Chiayi, Taiwan

**Keywords:** congenital heart disease, device migration, Konar-MFO, transcatheter closure, ventricular septal defect

## Abstract

Late device migration following transcatheter perimembranous ventricular septal defect (VSD) closure is exceedingly uncommon, as most cases of embolization occur within the first hours to weeks after implantation. In the present case, migration was observed 18 months post-procedure, representing an unusually late presentation. Notably, surgical retrieval demonstrated complete circumferential endothelialization of the device, confirming true long-term tissue incorporation before subsequent erosion and detachment. Furthermore, serial early and mid-term imaging consistently showed a stable device position, making early malposition or technical error an unlikely cause. This constellation of findings underscores the rarity of very late mechanical destabilization after an initially successful device closure.

## Introduction

Ventricular septal defects (VSDs) are the most prevalent form of congenital heart disease (1, 2). Among the various subtypes, perimembranous ventricular septal defects are the most commonly observed (1). In recent years, transcatheter VSD closure has demonstrated favorable clinical outcomes and has been increasingly performed in many countries for carefully selected patients (1, 3). This approach has gained popularity owing to its minimally invasive nature, faster recovery, shorter hospital stays, and avoidance of complications associated with cardiopulmonary bypass (2, 4).

The LifeTech™ Multifunctional Occluder (Konar-MFO) is a novel device that received Conformité Européenne (CE) approval in 2018 (4). Recent studies have demonstrated that transcatheter closure of membranous and muscular VSDs using the Konar-MFO device is feasible and effective in selected patients, with high procedural success rates and a low incidence of early complications (5). Previous reports have likewise suggested that transcatheter closure of membranous and muscular VSDs using the Konar-MFO device is safe and efficacious in appropriately selected cases (1, 4). Owing to its design, the device has been shown to minimize injury to adjacent cardiac structures and to reduce the incidence of rhythm-related complications when compared with earlier occluder systems (4). Overall, the Konar-MFO device has been associated with a high procedural success rate and a low complication profile (4).

The most relevant complications of transcatheter VSD closure include residual shunt, device-related arrhythmia, vascular complications, hemolysis, valvular dysfunction, and device embolization (2, 3). In a pooled analysis, the estimated incidence of device embolization following transcatheter VSD closure was reported to be approximately 0.4% (4). Most embolization events occur during the procedure or in the early post-procedural period and are commonly attributed to underestimation of VSD size or suboptimal device selection (3, 4). In contrast, very late device migration after transcatheter closure of perimembranous VSDs has rarely been reported. Herein, we report a rare case of very late migration of a Konar-MFO device occurring 18 months after transcatheter closure of a perimembranous VSD in a pediatric patient, highlighting a potential mechanism of late device instability despite initially successful implantation.

## Case description

**Figure 1:**
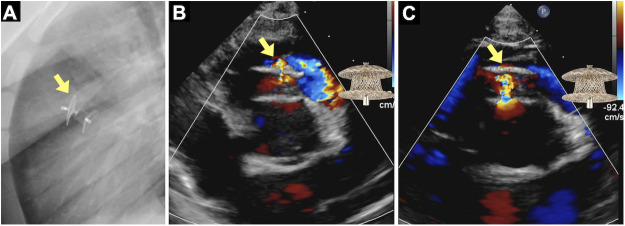
Early post-implantation imaging **(A)** chest radiography obtained on postoperative day 1 demonstrating appropriate orientation and alignment of the konar-MFO device (arrow). **(B)** Transthoracic echocardiography (short-axis view) on postoperative day 1 showing a well-seated device (arrow) with a trivial intradisc residual shunt. **(C)** Twelve-month follow-up echocardiography confirming stable device position (arrow) without interval change. The previously noted trivial intradisc residual shunt had decreased and was no longer detectable by Doppler interrogation. (A schematic illustration of the occluder device is superimposed on the echocardiographic image to demonstrate device angulation).

A 4-year-old boy was referred for further evaluation and treatment due to a ventricular septal defect (VSD). Transthoracic echocardiography demonstrated a defect located slightly anteriorly compared with a typical perimembranous VSD, but not fully consistent with the classic outlet or subarterial type. Based on these anatomical features, the defect was classified as a perimembranous VSD with outlet extension. Mild right coronary cusp prolapse toward the defect was also noted. The VSD size was measured using both transthoracic echocardiography and left ventricular angiography. The distance between the VSD and the aortic valve (aortic rim) measured approximately 5.5 mm on echocardiography. The VSD diameter was 4.4 mm by echocardiography and 3.5 mm by angiography. After evaluating the VSD size and its anatomical relationship to the aortic valve, the transcatheter closure procedure was subsequently performed. After femoral arterial and venous access was obtained, a guidewire was advanced retrogradely from the aorta into the left ventricle and across the VSD into the right ventricle (RV) and pulmonary artery, where it was subsequently snared to create an arteriovenous loop. A delivery sheath was then advanced antegradely from the femoral vein over the arteriovenous loop across the defect into the ascending aorta. The device size was selected based on the maximal diameter obtained from these imaging modalities, with particular attention to the aortic valve and surrounding rim, to ensure optimal occlusion without affecting adjacent structures. A 7/5-mm Konar-MFO ventricular septal defect occluder (MFO; Lifetech, Shenzhen, China) was then deployed, with the left disc and truncated cone released in the ascending aorta and subsequently positioned against the interventricular septum. The device waist was carefully seated within the septal defect, followed by deployment of the right disc in the RV. The angiographic device waist after deployment measured approximately 4.6 mm. Final angiography and transthoracic echocardiography confirmed appropriate device position. The implantation was optimal, with trivial residual shunt and trivial aortic regurgitation. Chest radiography and transthoracic echocardiography performed on the day after the procedure confirmed appropriate device position (Figures 1A,B). Serial echocardiographic follow-up was performed at regular intervals during the first year after the procedure, consistently demonstrating stable device positioning without evidence of device deformation. At the 12-month follow-up visit (the fifth follow-up examination), echocardiography continued to show a stable device position without deformation. Mild aortic regurgitation remained unchanged, and the previously noted trivial intradisc residual shunt had decreased and was no longer detectable by Doppler interrogation (Figure 1C).

**Figure 2:**
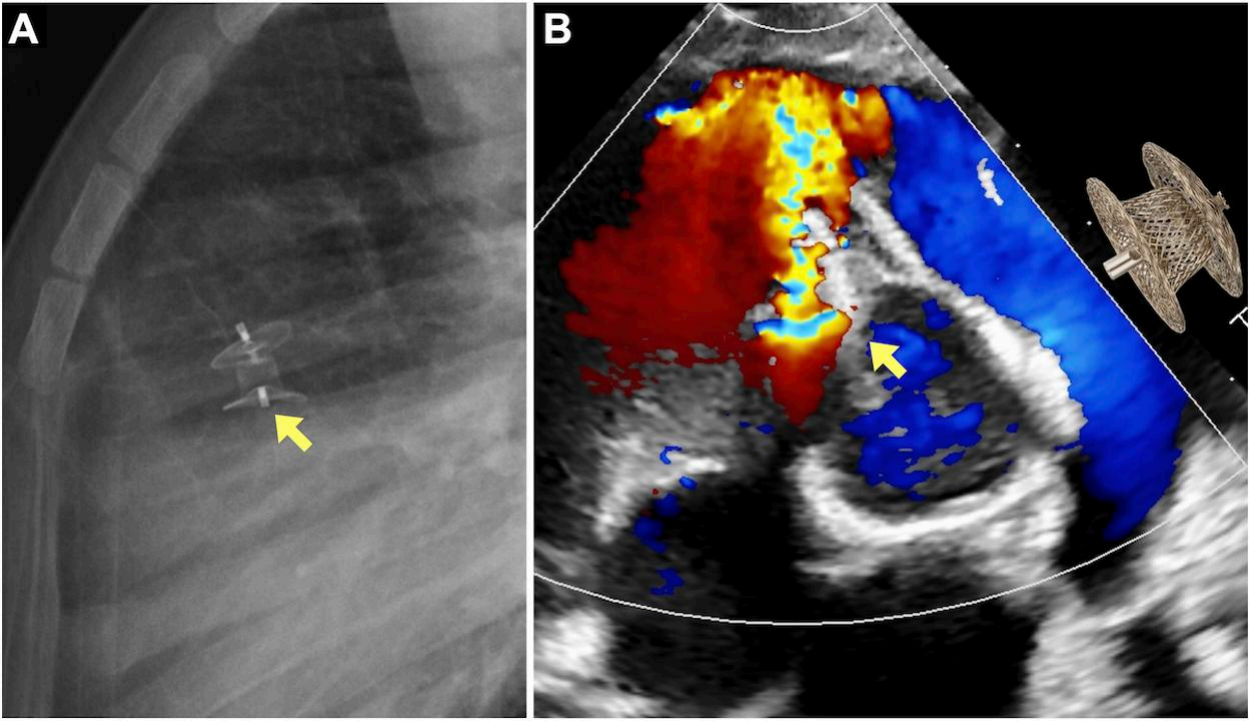
Eighteen-Month surveillance imaging demonstrating late device migration **(A)** chest radiograph revealing altered device angulation (arrow) compared with prior studies. **(B)** Transthoracic echocardiography (short-axis view) showing migration of the occluder (arrow) into the right ventricular outflow tract (RVOT), accompanied by an increased left-to-right shunt but no Doppler evidence of RVOT obstruction. (A schematic illustration of the occluder device is superimposed on the echocardiographic image to demonstrate device angulation).

At about 18 months after device implantation, a louder systolic murmur prompted re-evaluation. Chest radiography showed a change in device angulation (Figure 2A), and transthoracic echocardiography revealed migration of the occluder into the right ventricular outflow tract (RVOT) with increased left-to-right shunt (Figure 2B). Computed tomography demonstrated the device near the pulmonary valve without obstruction. The patient remained asymptomatic, and there was no history of any external traumatic events prior to the detection of device migration.

**Figure 3:**
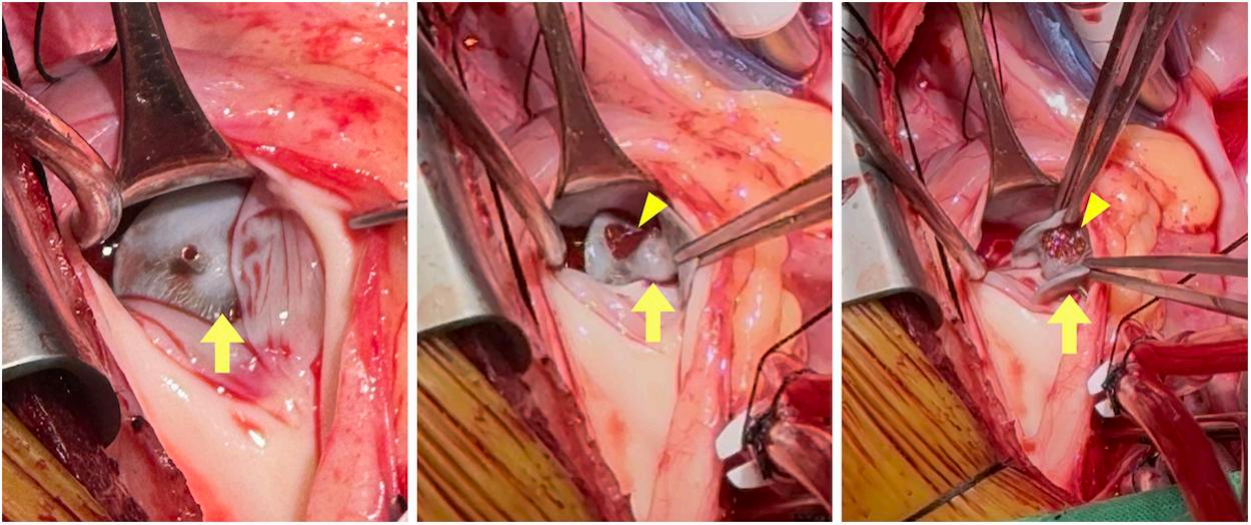
Operative findings following surgical retrieval. Intraoperative inspection revealed near-complete circumferential endothelialization of the ventricular side of the occluder (arrow). A focal tear (arrowhead) at the device-septal interface was identified.

Surgical exploration revealed extensive endothelialization of the occluder (Figure 3), along with a focal tear at the device-septal interface, indicating stable incorporation prior to late detachment. There was no gross evidence of inflammatory infiltration or extensive tissue necrosis at the time of surgery. The VSD was repaired surgically, and recovery was uneventful.

## Discussion

Several mechanisms may explain this unusually late migration. Previous studies have suggested that interaction between the aortic valve cusp and perimembranous VSD occluders may generate repetitive mechanical stress at the device–septal interface, potentially predisposing to late erosion or device instability, particularly in the presence of aortic valve prolapse (6). Although the Konar-MFO device is specifically designed with a soft and flexible nitinol wire mesh that conforms to the plane of the aortic valve without impairing valvular coaptation, such flexibility may also permit chronic cyclic deformation under continuous valvular motion (5).

Importantly, complete endothelialization does not necessarily confer permanent mechanical stability. Long-term observations have demonstrated that late adverse events may occur months to years after initially successful implantation, indicating that chronic mechanical trauma, compression, low-grade inflammation, and progressive scarring may evolve over time despite early procedural success (7). Although device migration is most commonly reported in the early post-procedural period and is usually attributed to inappropriate patient selection or technical factors (3, 5, 6), occluder migration remains a recognized cause of unplanned surgical intervention in large series (6).

**Figure 4:**
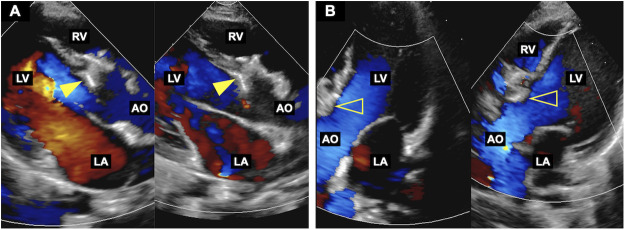
Cyclic deformation of the left disc skirt on serial echocardiography. **(A)** During diastole, the left disc skirt (arrowhead) is compressed by the posterior excursion of the right coronary cusp. **(B)** During systole, the skirt (open arrow) re-expands, restoring its normal contour.

In the present case, the patient had right coronary cusp (RCC) prolapse, which may have imposed chronic cyclic traction on the device-septal interface, promoting micro-erosion. The presence of outlet extension and the proximity of the defect to the aortic valve may have further increased the susceptibility of the device-septal interface to repetitive mechanical interaction. Although the Konar-MFO device is highly flexible (1) and adapts to complex anatomy, this flexibility may also allow greater deformation under dynamic forces. Dynamic imaging was pivotal in elucidating this mechanism: although initially well positioned, follow-up imaging revealed intermittent diastolic compression of the left disc skirt by posterior motion of the RCC (Figure 4). This repeated diastolic compression-systolic recoil pattern suggests a chronic cyclic load on the device skirt, potentially predisposing to structural fatigue and focal weakening. Over time, such fatigue-related changes may have resulted in micro-abrasion and progressive erosion (2), which, together with persistent anterior forces, could have contributed to displacement of the fully endothelialized device into the RVOT. Device undersizing was also considered as a potential contributing factor. During the procedure, implantation of a larger device was briefly considered because of a trivial residual shunt. However, angiography after deployment demonstrated that the device waist measured approximately 4.6 mm, suggesting an appropriate device size. In addition, a larger device might elongate under compressive forces and potentially increase interaction with the aortic valve, thereby posing a greater risk of valvular interference. Therefore, implantation of a larger device was not selected.

Hemodynamic forces related to residual shunting were also considered as a potential contributing mechanism but were considered unlikely to play a major role. Early post-procedural imaging demonstrated only a trivial intradisc residual shunt, which progressively decreased during follow-up and eventually became undetectable by Doppler interrogation. Therefore, shunt-related flow forces were unlikely to have contributed significantly to the late device displacement observed in this case.

Intraoperative findings revealed a focal tear at the device–septal interface without gross evidence of inflammatory infiltration or extensive tissue necrosis. Repetitive cyclic interaction between the occluder and the septal tissue may have contributed to progressive tissue weakening over time, potentially predisposing to late device instability and migration. We did not perform histopathological examination of the surrounding tissue to evaluate potential erosion-related injury, which represents a limitation of this report.

Device embolization after perimembranous VSD closure is uncommon and typically occurs during the procedure or within the early post-procedural period (hours to days) (1, 4). Although previous reports, such as Haddad et al. (8), have described late embolization occurring in the mid-term follow-up period (3-6 months post implantation), the device was partially endothelialized at the time of displacement. In contrast, the present case demonstrates very late migration at about 18 months despite complete endothelialization and stable early imaging, indicating that procedural error was unlikely. This observation highlights that late migration may still occur regardless of whether endothelialization is complete, and that subtle cyclic deformation detected on dynamic imaging, rather than clinical symptoms, may be the earliest indicator of future instability and late complications.

Accordingly, prospective long-term follow-up incorporating dynamic imaging assessment may be valuable in defining the durability and safety of transcatheter VSD closure, even in patients with an initially successful outcome (7).

### Teaching point

Subtle cyclic deformation of a VSD occluder on dynamic imaging may precede late device instability, underscoring the need for long-term imaging follow-up even after successful implantation.

## Data Availability

The original contributions presented in the study are included in the article/Supplementary Material, further inquiries can be directed to the corresponding author.
